# Effect of Pretreatment Platelet Parameters on Survival in Limited Disease Small Cell Lung Cancer

**DOI:** 10.31557/APJCP.2019.20.6.1879

**Published:** 2019

**Authors:** Abdullah Sakin, Nurgul Yasar, Serdar Arici, Cumhur Demir, Caglayan Geredeli, Ferdi Aksaray, Selver Isik, Sener Cihan

**Affiliations:** 1 *Department of Medical Oncology, Yuzuncu Yil University Medical School, Van, *; 2 *Department of Medical Oncology, *; 3 *Department of Radiation Oncology, University of Health Sciences, Okmeydani Training and Research Hospital, Istanbul, *; 4 *Department of Medical Oncology, University of Healt Sciences, Erzurum Bolge Training and Research Hospital, Erzurum, Turkey. *

**Keywords:** Small cell lung carcinoma, limited disease, chemoradiotherapy, total platelet count, survival

## Abstract

**Background:**

The aim of this study was to investigate the effect of platelet parameters before concurrent chemoradiotherapy (CCRT) on survival of patients with limited disease small cell lung cancer (LD-SCLC).

**Methods:**

This study consisted of patients who received CCRT due to LD-SCLC in the oncology clinic between 1997-2017. Examined platelet parameters included total platelet count (TPC), mean platelet volume, platelet distribution width, and platelet-lymphocyte ratio. The cut-off value for TPC was determined as 306x10^9^/U (sensitivity: 62%, specificity: 75.5%), where patients below or equal to this level was classified as Group I, and those above as Group II.

**Results:**

The study included 90 patients whose mean age was 59 years (range: 42-83) and male ratio was 80.0% (n=72). Near three-fourths of patients (74.4%) were at clinical stage III. Among stage I-II patients, mOS was found as 126 months for Group I whereas it had not been reached in Group II (p=0.158). Stage III patients showed significantly lower mOS for Group 1 (16 [range: 14.1-17.8] months) compared to that in Group 2 (19.0 [range: 15.6-62.8] months; p=0.002). In multivariate analysis, Eastern Cooperative Oncology Group performance score (p=0.003), clinical stage (p<0.001), prophylactic cranial irradiation (p=0.004), and TPC (p=0.031) was determined as the most significant factors affecting survival.

**Conclusion:**

Our study suggests association of high baseline levels of TPC to improved survival in patients scheduled to undergo CCRT for LD-SCLC. Considering easiness and universal availability of TPC measurement, potential utilization of this biomarker may be promising to predict survival, albeit requiring validation by further well-designated prospective studies.

## Introduction

The most common cause of cancer-related deaths worldwide is the lung cancer. Approximately 1.8 million patients were diagnosed with lung cancer and near 1.6 million lung cancer-related deaths occurred across the globe in 2012 (Siegel et al., 2018).

Small cell lung cancer (SCLC) accounts for about 15% of all lung cancers. It is clinically different from non-small cell lung cancer (NSCLC) because of its rapid progression and early development of metastases (Gaspar et al., 2005; Silvestri et al., 2013). Only 5% of all SCLC patients are diagnosed at the limited disease (LD) stage of the disease; the remaining patients get the diagnosis at the extensive disease (ED) stage. Highly greater probability of ED at the time of diagnosis can be attributed to the high degree of aggressive biology, rapid tumor progression, absence of symptoms in the early stage, and lack of well-established screening programs. Chemoradiotherapy (CRT) remains the standard treatment modality in LD-SCLC (Chen et al., 2010; Varlotto et al., 2011). On the other hand, potential prognostic factors are still unclear in this patient subset.

Platelets are responsible for initiating hemostatic mechanisms that abolish vascular endothelial injury (Smyth et al., 2009). Several recent studies have reported that platelets were associated with development and progression of malignancy (Riedl et al., 2014; Franco et al., 2015). Activated platelets were suggested to promote cancer cell growth, angiogenesis, and invasion (Buergy et al., 2012). Furthermore, association of some platelets indices were reported to prognosis in various cancers, including NSCLC, breast cancer, colorectal cancer, gastric cancer, pancreatic cancer, and laryngeal cancer. Several of these parameters, including total platelet count (TPC), mean platelet volume (MPV), platelet distribution width (PDW), and platelet-lymphocyte ratio (PLR) can be easily tested (Song et al., 2017; Zhang et al., 2017a; Zhang et al., 2017b). Nevertheless, the findings of studies reporting the association of platelet counts and other platelet indices to the presence and progression of cancer are still controversial and suggest the need for clarification (Monreal et al., 1998; Liu et al., 2017; Pedrazzani et al., 2017; Zhang et al., 2019). 

This study aimed to investigate the effect of selected pretreatment platelet indices on survival of patients with LD-SCLC.

## Materials and Methods

*Patients*

This retrospective study included patients who received concurrent chemoradiotherapy (CCRT) due to LD-SCLC in the oncology clinic between 1997-2017. Staging of patients were performed according to findings of pre-treatment fluorodeoxyglucose positron emission tomography (PET-CT), computed tomography (CT), and cranial magnetic resonance imaging (MRI). Tumor size was calculated by measuring the largest tumor diameter in CT. Exclusion criteria were malignancy other than SCLC, or those having ED-SCLC, autoimmune disease, history of aspirin or any immunosuppressive drug use, being under the age of 18, or whose data were not accessible. In our hospital, SCLC staging is performed based on PET-CT and cranial MRI findings, and pre-treatment clinical, pathological, and laboratory data are recorded. Ensuring a 10-12 hours of fasting, samples for blood tests are routinely taken from antecubital vein by establishing mild venous stasis at upper arm in patients who apply to oncology clinic before treatment. Blood samples for biochemical parameters are taken into the anticoagulant-free gel tubes, and those for complete blood counts into ethylenediamine tetraacetate-containing tubes. Complete blood count parameters are examined in hemogram autoanalyser (Mindray, China). Biochemical parameters are tested in autoanalyser (Beckman Coulter, USA) using colorimetric method. Based on Eastern Cooperative Oncology Group performance score (ECOG PS) and comorbidities, patients are treated with concomitant CRT of two cycles with platinum [cisplatin (60-75 mg/m2) or carboplatin (AUC 5)] + etoposide (100 mg/m2 for 1-3 days) at 28-day intervals, followed with two to four cycles of the same chemotherapeutic regimen at 21-day intervals. Radiotherapy is administered as a total of 60 Gy, consisting of 30 fractions as 2 Gy per day.

**Figure 1:**
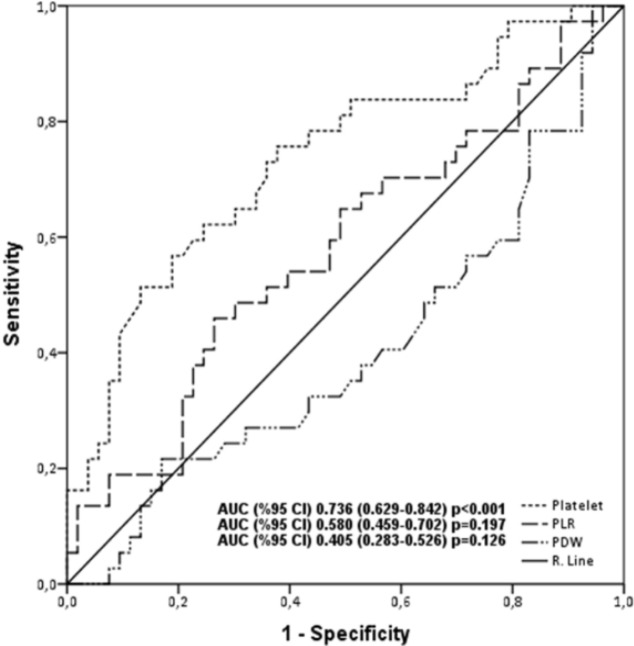
ROC Curve Analyses for OS; platelets are represented by the line with AUC = 0.736 (95% CI, 0.629–0.842; p<0.001) with a sensitivity of 62.2% and a specificity of 75.5% (306x103). AUC, the area under the curve, PDW, platelet distribution width, PLR, platelet-to-lymphocyte ratio

Data collection: Data on the age, gender, smoking status, ECOG PS, history of superior vena cava syndrome (SVCS), clinical tumor stage, chemotherapy regimen, cycle count, history of prophylactic cranial irradiation (PCI), presence of recurrence/metastasis, and survival status were obtained from medical records. In addition, initial laboratory values immediately after diagnosis were collected: creatinine, aspartate aminotransferase (AST), alanine aminotransferase (ALT), alkaline phosphatase (ALP), gamma glutamyl transferase (GGT), lactate dehydrogenase (LDH), sodium, potassium, calcium, albumin, white blood cell (WBC) count, red blood cell (RBC) count, hemoglobin (Hb), hematocrit (Hct), mean corpuscular volume (MCV), total platelet count (TPC), total neutrophil count (TNC), total lymphocyte count (TLC), and total monocyte count (TMC). Neutrophil-lymphocyte ratio (NLR) was obtained by dividing the TNC by TLC. PLR was obtained by dividing TPC by TLC. Monocyte-lymphocyte ratio (MLR) was obtained by dividing the TMC by TLC. Patients were further stratified by their age as<65 years and ≥65 years. Patients’ ECOG PS were grouped as either 0-2 or 3-4. Receiver operating characteristics (ROC) curves were formed for overall survival (OS) using TPC, PDW, and PLR data at diagnosis. The areas under the curve (AUCs) were detected as 0.736 (95% CI 0.629-0.842, p<0.001), 0.580 (95% Cl 0459-0702, p=0.197), and 0.405 (95% Cl 0.283-0,526, p=0.126), respectively. The cut-off value for TPC was determined as 306x10^9^/U with a sensitivity of 62% and a specificity of 75.5% (Figure 1). Patients having a TLC of ≤306x10^9^/U were categorized as Group I and those having a TLC of >306x10^9^/U were as Group II. OS was calculated as time from the diagnosis to the date of last follow-up or death. The study was performed in accordance with the declaration of Helsinki and was reviewed and approved by the Ethics Committee of the University of Health Sciences, Okmeydani Training and Research Hospital (26.2.18).

Statistical analysis: Statistical Package for the Social Sciences 22.0 for Windows software (Armonk NY, IBM Corp, 2013) was used for the statistical analysis. Descriptive statistics were presented as the mean, standard deviation, minimum, and maximum values for numerical variables. The comparison of the rates between the groups was performed by chi-square analysis. Numerical variable between two independent groups were analyzed with student t-test in case of normal distribution and with Mann Whitney U test if else. Monte Carlo simulation was applied if conditions could not be met. Survival analyses were performed with Kaplan-Meier Analysis. Determinant factors were examined with cox regression. Backward stepwise model was used with parameters having a p-value below 0.100. An overall 5% alpha error level was used to infer statistical significance.

**Table 1:** Demographic and Clinical Characteristics of the Study Population

		All patient		Group 1		Group 2		
		n	%	n	%	n	%	p
Gender	Female	18	20	8	14.8	10	27.8	0.132
	Male	72	80	46	85.2	26	72.2	
Age group	<65 years	70	77.8	41	75.9	29	80.6	0.605
	≥65 years	20	22.2	13	24.1	7	19.4	
Smoking status	No	4	4.4	1	1.9	3	8.3	0.144
	Yes	86	95.6	53	98.1	33	91.7	
ECOG PS	0-1-2	77	85.6	44	81.5	33	91.7	0.178
	3-4	13	14.4	10	18.5	3	8.3	
SVCS	No	84	93.3	51	94.4	33	91.7	0.605
	Yes	6	6.7	3	5.6	3	8.3	
Clinical stage	I+II	23	25.6	11	20.4	12	33.3	0.167
	III	67	74.4	43	79.6	24	66.7	
Chemotherapy regimen	Cisplatin + etoposide	82	91.1	49	90.7	33	91.7	0.879
	Carboplatin + etoposide	8	8.9	5	9.3	3	8.3	
PCI	No	40	44.4	28	51.9	12	33.3	0.083
	Yes	50	55.6	26	48.1	24	66.7	
Relapse/metastasis	No	19	21.1	9	16.7	10	27.8	0.206
	Yes	71	78.9	45	83.3	26	72.2	
Final status	Alive	37	41.1	14	25.9	23	63.9	<0.001
	Exitus	53	58.9	40	74.1	13	36.1	
Age (years)	Mean (Min-Max)	59.0 (42-83)		60.0 (42-77)		58.0 (46-83)		0.166
Smoking (pack-years)	Mean ± SD (Min-Max)	57.8 ± 34.7 (0-180)		57.6 ± 30.8 (0-180)		58.0 ± 40.3 (0-160)		0.67
Tumor size (cm)	Mean ± SD (Min-Max)	5.8 ± 2.6 (2.0-15.0)		5.4 ± 2.2 (2.0-10.5)		6.5 ± 3.1 (2.0-15.0)		0.131
The number of cycles of chemotherapy	Mean ± SD (Min-Max)	4.9 ± 1.1 (2-6)		4.8 ± 1.2 (2-6)		5.1 ± 1.0 (3-6)		0.268

ECOG PS, Eastern Cooperative Oncology Group Performance Status; PCI, prophylactic cranial irradiation; SVCS, superior vena cava syndrome.

**Table 2:** Baseline Laboratory Parameters of the Study Population

	All patient		Group 1		Group 2		
	n	Mean ± SD (Min-Max)	n	Mean ± SD (Min-Max)	n	Mean ± SD (Min-Max)	p
Creatinine (mg/dL)	84	0.8 ± 0.2 (0.3-1.5)	49	0.8 ± 0.2 (0.5-1.5)	35	0.8 ± 0.2 (0.3-1.3)	0.114
AST (U/L)	79	22.4 ± 12.6 (9-79)	47	24.1 ± 14.3 (10-79)	32	19.8 ± 9.0 (9-60)	0.24
ALT (U/L)	79	22.1 ± 15.3 (7-104)	47	21.4 ± 13.0 (7-64)	32	23.3 ± 18.4 (7-104)	0.869
GGT (U/L)	58	52.7 ± 77.6 (8-467)	33	61.3 ± 99.3 (8-467)	25	41.4 ± 30.3 (10-153)	0.081
LDH (U/L)	57	259.3 ± 119.4 (120-671)	31	259.2 ± 101.9 (120-560)	26	259.5 ± 139.5 (120-671)	0.718
ALP (U/L)	57	114.1 ± 88.7 (15-546)	33	119.3 ± 97.8 (35-546)	24	107.1 ± 75.9 (15-340)	0.821
Sodium (mmol/L)	43	139.3 ± 3.3 (130-146)	25	139.0 ± 3.7 (130-145)	18	139.7 ± 2.6 (134-146)	0.804
Potassium (mmol/L)	41	4.5 ± 0.4 (3.8-5.5)	23	4.5 ± 0.5 (3.8-5.5)	18	4.4 ± 0.4 (3.9-5.2)	0.252
Calcium (mg/dL)	40	9.5 ± 0.4 (8.5-10.7)	25	9.6 ± 0.5 (8.5-10.7)	15	9.5 ± 0.2 (9.0-9.9)	0.474
WBC (10^9^/U)	90	8.9 ± 3.5 (2.5-25.5)	54	8.3 ± 3.2 (2.5-21.1)	36	9.9 ± 3.9 (4.4-25.9)	0.011
Rbc (10^9^/U)	90	4.5 ± 0.6 (3.0-6.4)	54	4.6 ± 0.7 (3.0-6.4)	36	4.4 ± 0.4 (3.4-5.2)	0.076
Hb (g/dL)	90	12.9 ± 1.7 (9.3-17.3)	54	13.3 ± 1.8 (9.3-17.3)	36	12.3 ± 1.5 (9.7-15.2)	0.004
Hct (%)	90	39.2 ± 5.0 (27.6-49.5)	54	40.3 ± 5.2 (27.6-49.5)	36	37.5 ± 4.2 (30.1-46.0)	0.005
MCV (fL)	90	86.7 ± 6.8 (64.1-102.2)	54	87.6 ± 6.5 (67.1-102.2)	36	85.3 ± 7.2 (64.1-98.6)	0.12
TPC (10^9^/U)	90	302.9 ± 128.3 (38.8-798.0)	54	222.4 ± 56.8 (38.8-305.0)	36	423.6 ± 109.7 (307-798)	<0.001
PDW (%)	90	14.8 ± 7.9 (8.2-64.4)	54	16.0 ± 9.8 (8.2-64.4)	36	13.0 ± 3.0 (8.2-17.4)	0.124
MPV (fL)	90	8.9 ± 1.5 (5.3-13.1)	54	9.2 ± 1.6 (5.8-13.1)	36	8.4 ± 1.4 (5.3-10.4)	0.04
RDW (%)	81	14.5 ± 1.7 (12.0-19.4)	48	14.5 ± 1.9 (12.1-19.4)	33	14.4 ± 1.5 (12.0-19.2)	0.893
TNC (10^9^/U)	90	6.1 ± 3.2 (1.6-23.6)	54	5.5 ± 2.8 (1.6-18.5)	36	6.8 ± 3.6 (2.4-23.6)	0.011
TLC (10^9^/U)	90	1.9 ± 0.8 (0.4-3.4)	54	1.9 ± 0.8 (0.4-3.4)	36	2.0 ± 0.7 (0.5-3.4)	0.424
TMC (10^9^/U)	90	0.7 ± 0.3 (0.1-2.0)	54	0.6 ± 0.3 (0.1-1.4)	36	0.7 ± 0.3 (0.4-2.0)	0.168
NLR	90	3.5 ± 2.8 (1.1-20.1)	54	3.3 ± 2.5 (1.1-16.8)	36	3.9 ± 3.3 (1.1-20.1)	0.236
PLR	90	180.4 ± 120.8 (19.4-761.1)	54	138.5 ± 77.6 (19.4-455.6)	36	243.2 ± 145.6 (100.6-761.1)	<0.001
MLR	90	0.4 ± 0.3 (0.1-2.0)	54	0.4 ± 0.2 (0.1-1.3)	36	0.4 ± 0.3 (0.2-2.0)	0.595

ALT, alanine aminotransferase; AST, aspartate aminotransferase; ALP, alkaline phosphatase; Hb, hemoglobin; Hct, hematocrit; GGT, gamma glutamyl transferase; LDH, lactate dehydrogenase; MCV, mean corpuscular volume; MLR, monocyte-to-lymphocyte ratio; MPV, mean platelet volume; NLR; neutrophil-to-lymphocyte ratio; PDW, platelet distribution width; PLR, platelet-to-lymphocyte ratio; RBC, red blood cell; RDW, red cell distribution width; TLC, total lymphocyte count; TMC, total monocyte count; TNC, total neutrophil count; TPC, total platelet count; WBC, white blood cell.

**Table 3:** Univariate Analysis for OS

Variable		HR	95% CI	p
Gender	male vs. female	1.512	0.711-3.215	0.283
Age	years	1.045	1.013-1.078	0.006
Smoking status	no vs. yes	1.824	0.438-7.599	0.409
ECOG PS	2-4 vs. 0-1	4.393	2.258-8.545	<0.001
Clinical stage	stage 3 vs. 1-2	6.167	2.579-14.746	<0.001
PCI	yes vs. no	0.327	0.188-0.570	<0.001
The number of cycles of chemotherapy	≤4 vs. >4	0.483	0.277-0.842	0.01
Smoking	pack-years	0.999	0.992-1.006	0.826
Tumor size	cm	0.92	0.820-1.032	0.156
Creatinine	mg/ dL	1.661	0.415-6.647	0.473
AST	U/L	1.008	0.987-1.029	0.463
ALT	U/L	0.999	0.977-1.022	0.954
GGT	U/L	1.003	1.000-1.007	0.081
LDH	U/L	1	0.997-1.002	0.81
ALP	U/L	0.999	0.994-1.004	0.689
Sodium	mmol/L	0.963	0.855-1.085	0.534
Potassium	mmol/L	2.602	0.998-6.785	0.055
Calcium	mg/ dL	0.556	0.181-1.710	0.306
WBC	10^9^/U	0.971	0.896-1.053	0.478
RBC	10^9^/U	0.936	0.582-1.505	0.785
Hb	g/dL	1.057	0.905-1.236	0.483
Hct	%	1.017	0.962-1.074	0.556
MCV	fL	1.025	0.983-1.070	0.24
TPC	10^9^/U	0.996	0.994-0.999	0.002
PDW	%	1.033	1.003-1.065	0.031
MPV	fL	1.092	0.917-1.300	0.325
RDW	%	1.102	0.921-1.318	0.29
TNC	10^9^/U	0.971	0.891-1.058	0.5
TLC	10^9^/U	1.031	0.700-1.517	0.878
TMN	10^9^/U	1.054	0.401-2.770	0.915
NLR		0.979	0.893-1.075	0.662
PLR		0.997	0.994-1.000	0.028
MLR		1.186	0.410-3.432	0.753

ALT, alanine aminotransferase; AST, aspartate aminotransferase; ALP, alkaline phosphatase; ECOG PS, Eastern Cooperative Oncology Group; Hb, hemoglobin; Hct, hematocrit; GGT, gamma glutamyl transferase; LDH, lactate dehydrogenase; MCV, mean corpuscular volume; MLR, monocyte-to-lymphocyte ratio; MPV, mean platelet volume; NLR; neutrophil-to-lymphocyte ratio; PCI, prophylactic cranial irradiation; PDW, platelet distribution width; PLR, platelet-to-lymphocyte ratio; RBC, red blood cell; RDW, red cell distribution width; SVCS, superior vena cava syndrome; TLC, total lymphocyte count; TMC, total monocyte count; TNC, total neutrophil count; TPC, total platelet count; WBC, white blood cell.

**Table 4:** Multivariate Analysis for OS

Variable		HR	95% CI	p
ECOG PS	2-4 vs. 0-1	2.849	1.441-5.633	0.003
Clinical stage	3 vs. 1-2	5.542	2.277-13.489	<0.001
PCI	Yes vs. No	0.447	0.248-0.805	0.004
TPC	10^9^ /U	0.997	0.995-0.999	0.031

ECOG PS, Eastern Cooperative Oncology Group; PCI, prophylactic cranial irradiation; TPC, total platelet count.

**Figure 2:**
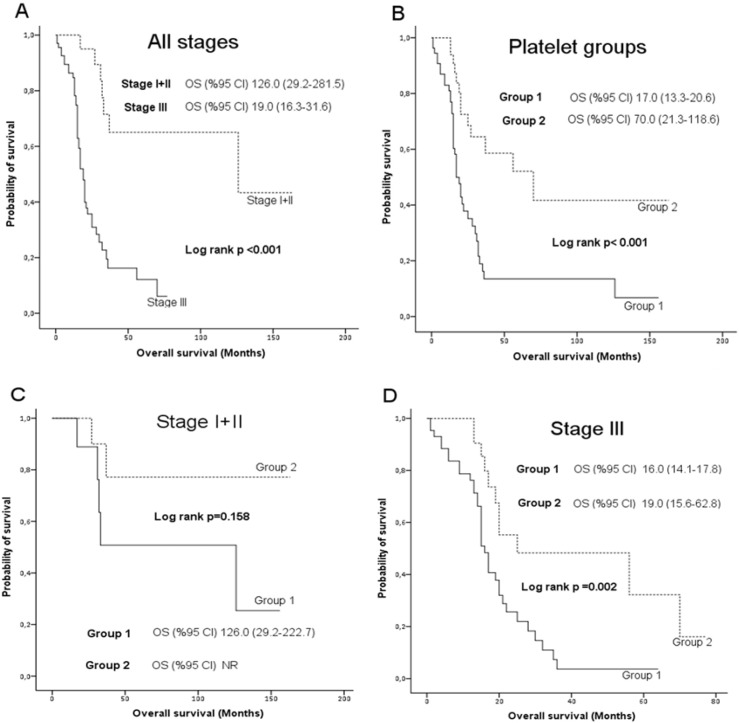
Overall Survival by (A) Clinical Stages, (B) Platelet Groups; (C) Platelet Groups in Stage I+II, and (D) Platelet Groups in Stage III

## Results

The study included 90 patients whose mean age was 59 years (range: 42-83), and male cases constituted 80.0% (n=72). Twenty patients (22.2%) were ≥65 years old. Most of patients (95.6%) had history of smoking. Thirteen patients (14.4%) had ECOG PS of ≥2. SVCS was present at time of diagnosis in six patients (6.7%). A quarter of patients (25.6%) were at clinical stage I or II. Patients received mostly (91.1%) cisplatin + etoposide protocol, with a mean number of 4.9 cycles overall. PCI was performed in 50 patients (55.6%). During the follow-up, 71 (78.9%) patients had relapse/metastasis and 53 (58.9%) patients had died. There were 54 patients (60.0%) in Group I and 36 patients (40.0%) in Group II. These study groups were found as similar in terms of age, gender, smoking status, ECOG PS, tumor localization, presence of SVCS, clinical stage, chemotherapy regimen, received cycle count, and relapse/metastasis rates (Table 1).

Group I had significantly higher levels of mean baseline Hb, Hct, and MPV (p=0.004, p=0.005, and p=0.040; respectively) and significantly lower levels of mean baseline WBC, TNC, and PLR (p=0.011, p=0.011, and p<0.001; respectively) compared to those in Group II. All other laboratory values did not significantly differ between these study groups (Table 2).

Median overall survival (mOS) was significantly higher in patients with clinical stage I or II compared to that in patients with clinical stage III (126 [range: 29.2-281.5] vs. 19 [range: 16.3-31.6] months, respectively; log rank p<0.001). mOS in Group I (17 [range: 13.3-20.6] months) was significantly lower than that in Group II (70 [range: 21.3-118.6] months; log rank p<0.001). Among stage I-II patients, mOS was found as 126 months for Group I whereas it had not been reached in Group II (log rank p=0.158). Stage III patients showed significantly lower mOS for Group I (16 [range: 14.1-17.8] months) compared to that in Group II (19.0 [range: 15.6-62.8] months; log rank p=0.002), (Figure 2).

Univariate analysis showed the age, ECOG PS, clinical stage, PCI, number of chemotherapy cycles, TPC, PDW, and PLR as the significant factors that affected survival (p=0.006, p<0.001, p<0.001, p<0.001, p=0.010, p=0.002, p=0.031, and p=0.028; respectively), (Table 3). In further multivariate analysis, Eastern Cooperative Oncology Group performance score (p=0.003), clinical stage (p<0.001), prophylactic cranial irradiation (p=0.004), and TPC (p=0.031) was determined as the most significant factors affecting survival, (Table 4).

## Discussion

In this study, we investigated the effect of pre-treatment TPC, MPV, PDW, and PLR on survival of LD-SCLC patients managed with CRT. Interestingly, a higher TPC (>306x10^3^) was associated with improved survival in clinical stages I to III.

SCLC is known to have a more aggressive behavior than does NCSLC. In recent years, mean life expectancy has been improved owing to novel targeted therapy and immunotherapy modalities (Gadgeel et al., 2018; Uemura and Hida, 2018). However, survival in SCLC has not been much changed despite improvements in treatment options in the last 15 years (Gaspar et al., 2012). A recent study reported 2 months of improved survival with add-on atezolizumab to chemotherapy in ED-SCLC (Horn et al., 2018). All these indicate that the need for the development of simple and feasible prognostic factors in SCLC is extremely important. 

Inflammatory biomarkers reflect the responses of the host against malignant cells. Lately, there is a growing interest of the host’s inflammatory response against the tumor. Several studies have proposed NLR, PLR, and TPC as indicators of inflammatory conditions, and these indicators were described as both prognostic and predictive biomarkers in some cancers (Monreal et al., 1998; Forget et al., 2013; Lee et al., 2013; Pedrazzani et al., 2017; Song et al., 2017; Zhang et al., 2017a).

Currently, there are many articles discussing the effect of thrombocytosis on survival in NSCLC (Pedersen and Milman, 1996; Aoe et al., 2004; Tomita et al., 2009; Hong et al., 2015; Xie et al., 2015; Hong et al., 2018). However, there is scarce data on the prognostic importance of pre-treatment of TPC, MPV, PDW, and PLR in SCLC. Previous studies have reported mainly unfavorable or no impact of high platelet counts on survival of patients with NSCLC (Pedersen and Milman, 1996; Aoe et al., 2004; Tomita et al., 2009; Cakar et al., 2011).

Xie et al., (2015) in their study of 383 Chinese LD-SCLC patients reported low Hb, high PLR and NLR to be associated with poor prognosis; and determined important prognostic variables as PLR, age, smoking cessation, radiotherapy, chemotherapy, surgery, and PCI. Zhang et al., (2019) compared patients with high PLR (>152.1) to those with lower levels in their study regarding 286 Chinese LD-SCLC patients, and found mOS as 19.5 vs. 27.4 months, respectively; and concluded pre-treatment PLR as an independent variable for OS. 

Bernhardt et al., (2018) performed a study with 350 German LD-SCLC patients managed with CRT, where they reported no effect of NLR, age, Hb, and TPC on survival. The authors stated that multivariate analysis showed PCI, LDH, and CRP as independent factors affecting survival. Suzuki et al., (2019) in their study of 122 LD-SCLC patients who received CRT and followed up between 2002 and 2015, detected that lymphopenia was an independent poor prognostic factor.

Liu et al., (2017) determined NLR but not PLR as an independent prognostic factor in their study of 139 Chinese patients (55 LD-SCLC, 83 ED-SCLC). Hong et al., (2015) reported PLR, NLR, Hb, and MCV as significant prognostic factors in multivariate analysis in their study of 919 Chinese patients consisting of 552 LD-SCLC and 352 ED-SCLC cases, where the former group had a mOS of 11.8 months (Hong et al., 2015). Another study by Hong et al., (2018) among the 590 patients with LD-SCLC showed LDH, number of cycles of chemotherapy, and recurrence but not TPC as the independent factors affecting OS. Similarly, Deng et al. performed a study with 320 Chinese patients (122 LD-SCLC and 198 ED-SCLC), where they reported pre-treatment increased LDH and NLR but not PLR as an independent poor prognostic factor (Deng et al., 2017).

In our study, ECOG PS, clinical stage, PCI, and TPC were found to be independent factors affecting survival. All other laboratory parameters including NLR, PLR, MLR, TLC, MPV, and PDW were not associated with OS. The majority of studies about SCLC were performed on Chinese patients, among which treated with CRT had a mOS 11.2 to 27.4 months (Hong et al., 2015; Xie et al., 2015; Deng et al., 2017; Liu et al., 2017; Hong et al., 2018; Zhang et al., 2019). In our study, mOS was found as 25 months in all patients, including 126 months in stage I or II and 19 months in stage III. We did not measure the impact of ethnicity on our findings. For instance, German patients who received treatment with CRT had a mOS of 48 months (Kasmann et al., 2017).

Our study has several limitations. Although our study was performed only with LD-SCLC patients that received concomitant CRT with platinum + etoposide, we only included Turkish patients in a single-center setting with retrospective fashion. We cannot fully explain the mechanism of the effect of TPC on patients’ prognosis in LD-SCLC. We also do not know the degree of impact of ethnicity on our conclusions. We believe that our results warrant confirmation by studies involving more comprehensive studies with larger sample size.

In conclusion, our study suggests association of elevated TPC before treatment to prognosis in patients who received concomitant CRT with platinum + etoposide due LD-SCLC. Considering easiness and universal availability of TPC measurement, potential utilization of this biomarker may be promising to predict survival, albeit requiring validation by further well-designated prospective studies.

## Funding Source

None.

## Conflict-of-intereststatement

All authors declare no conflicts-of-interest related to this article. 
